# Establishing *Chlamydomonas reinhardtii* as an industrial biotechnology host

**DOI:** 10.1111/tpj.12781

**Published:** 2015-03-08

**Authors:** Mark A Scaife, Ginnie TDT Nguyen, Juan Rico, Devinn Lambert, Katherine E Helliwell, Alison G Smith

**Affiliations:** Department of Plant Science, University of CambridgeDowning Street, Cambridge, CB2 3EA, UK

**Keywords:** synthetic biology, industrial biotechnology, *Chlamydomonas reinhardtii*, metabolic engineering, rational design, transgene expression

## Abstract

Microalgae constitute a diverse group of eukaryotic unicellular organisms that are of interest for pure and applied research. Owing to their natural synthesis of value-added natural products microalgae are emerging as a source of sustainable chemical compounds, proteins and metabolites, including but not limited to those that could replace compounds currently made from fossil fuels. For the model microalga, *Chlamydomonas reinhardtii*, this has prompted a period of rapid development so that this organism is poised for exploitation as an industrial biotechnology platform. The question now is how best to achieve this? Highly advanced industrial biotechnology systems using bacteria and yeasts were established in a classical metabolic engineering manner over several decades. However, the advent of advanced molecular tools and the rise of synthetic biology provide an opportunity to expedite the development of *C. reinhardtii* as an industrial biotechnology platform, avoiding the process of incremental improvement. In this review we describe the current status of genetic manipulation of *C. reinhardtii* for metabolic engineering. We then introduce several concepts that underpin synthetic biology, and show how generic parts are identified and used in a standard manner to achieve predictable outputs. Based on this we suggest that the development of *C. reinhardtii* as an industrial biotechnology platform can be achieved more efficiently through adoption of a synthetic biology approach.

**Significance Statement:**

*Chlamydomonas reinhardtii* offers potential as a host for the production of high value compounds for industrial biotechnology. Synthetic biology provides a mechanism to generate generic, well characterised tools for application in the rational genetic manipulation of organisms: if synthetic biology principles were adopted for manipulation of *C. reinhardtii*, development of this microalga as an industrial biotechnology platform would be expedited.

## Microalgae Present Unique Features for Applied Research and Biotechnology

Microalgae are a polyphyletic group of unicellular eukaryotic organisms that can occupy both aquatic and terrestrial environments, adopting photosynthetic, heterotrophic or mixotrophic lifestyles. The enormous diversity in the algal lineages is due to their long evolutionary history (Dorrell and Smith, 2011). For instance the green algae, which are ancestral to land plants, diverged 1300 million years ago from the stramenopiles (Yoon *et al*., 2004), a group that encompasses several microalgal lineages of biotechnological relevance such as diatoms. As a result, microalgae exhibit wide variation in both cellular architecture and biosynthetic capacity, and thus present a number of general and unique features as research models and for commercial exploitation. Specifically, the unicellular physiology combined with rapid cell division and photosynthetic growth mean that microalgae can be more productive per unit land area than any plant system. In addition, using microalgae as feedstocks for low value, high volume products such as biofuels, provides the opportunity to avoid a number of environmental factors that currently affect biofuel production from crop plants (Chisti, 2007). These include issues surrounding land and (fresh)water usage, mono-culture, crop rotation, and input requirements: for example microalgae are able to sequester nitrogen and phosphorus from industrial waste streams, thus reducing the need for fertilisers. Nevertheless, many challenges remain to be overcome before it will be possible to cultivate algae on the very large scales needed for biofuel production (Scott *et al*., 2010; Klein-Marcuschamer *et al*., 2013). Instead, microalgae offer considerable potential for the production of low volume, high(er) value compounds, which are characteristically produced by industrial biotechnology (IB). Moreover, the photosynthetic lifestyle of microalgae means that they offer a more sustainable source of compounds compared to bacterial and yeast hosts used in conventional IB, since there is no need to provide fixed carbon.

Currently microalgae are exploited commercially for compounds that they make naturally, such as the pigments β-carotene and astaxanthin, and polymers alginate, carrageenan and agar for food products. Several research studies have attempted to enhance the production of value-added compounds, including bio-hydrogen from *Chlamydomonas reinhardtii* (Baltz *et al*., 2014; Xu *et al*., 2014), or omega-3 long chain fatty acids such as docosahexanoic acid (DHA) or eicosapentaenoic acid (EPA) from *Phaeodactylum tricornutum*. Although *P. tricornutum* naturally makes EPA and some DHA, levels are low and the ratio of the two fatty acids is suboptimal for some commercial applications. In addition, they are present mainly in membrane rather than storage lipids, making extraction inefficient. Hamilton *et al*. (2014) were able to increase levels of DHA in *P. tricornutum* eight-fold by heterologous expression of two fatty-acid modifying enzymes, a *Δ5-elongase* and an *acyl-CoA-dependent Δ6-desaturase*, from the green alga *Ostreococcus tauri* using a constitutive promoter. Another desirable feature of microalgae is their cellular complexity, which provides the opportunity to partition or compartmentalise biochemical reactions, something not possible in bacterial hosts. This can facilitate the provision of precursors, or provide intracellular sinks for target products. Indeed, there are already reports of the production of human therapeutic proteins including erythropoietin, interferon β proinsulin and immunoglobulin A in the chloroplast of *C. reinhardtii* (Rasala *et al*., 2010).

Whilst the many benefits afforded by microalgae are yet to be demonstrated at scale, these studies serve to raise the profile of microalgae research and development. The challenge now lies in the application of metabolic engineering strategies to enhance natural features of microalgae, making commercial exploitation economically viable (Klein-Marcuschamer *et al*., 2013). For example, in dense cultures required for industrial cultivation, light capture is limited. Reduction of the light-harvesting apparatus reduces light absorption per cell, increasing penetration and productivity within the culture (Polle *et al*., 2003; Cazzaniga *et al*., 2014). Other targets for optimisation include resource-use efficiency and auto-flocculation to facilitate harvesting. In parallel, to optimise and expand the level and range of novel products made by microalgae, it will be necessary to introduce new biosynthetic capabilities, remove competing pathways, and optimise the provision of precursor substrates and cofactors. Thus for microalgal IB to come of age, we need a suite of molecular biological tools with which to carry out sophisticated metabolic engineering.

For more than 20 years scientists have been able to redirect the metabolism of a host cell in a targeted manner through genetic manipulation, and this has been applied to across the tree of life, in both prokaryotes and eukaryotes, working with well characterised laboratory strains as well as novel environmental isolates. In the commercial sector, several organisms have been developed as robust IB hosts, including bacteria (*Escherichia coli*, *Bacillus subtilis*, *Corynebacterium glutamicum*, *Lactobacillus* spp.), and yeasts and fungi (*Saccharomyces cerevisiae*, *Aspergillus* spp.). These are used as production systems for compounds ranging from organic acids to pharmaceutical proteins (Goel *et al*., 2012; Buchholz and Collins, 2013). In spite of this success, metabolic engineering seldom demonstrates the design aspect that is so fundamental to engineering *per se*, since the development of bespoke solutions for each experimental system/product results in parallel development, and limits the transferability of constructs and knowledge. The advent of synthetic biology, whose principles involve a combination of standard parts, predictive modelling, and iterative design and testing, is just beginning to be exploited in the field of metabolic engineering, and holds out real promise for considerable advances in IB for production of compounds to reduce reliance on fossil fuels as feedstocks (Keasling, 2012; Yadav *et al*., 2012).

In this review, we present an overview of what has been achieved in terms of genetic manipulation of *C. reinhardtii*, and frame this in the context of bacterial and yeast model systems, drawing comparisons to illustrate current capabilities and limitations. We then discuss the relevance and timely nature of the development of synthetic biology concepts for metabolic engineering and how this represents a step change in capacity and predictive power. Finally we highlight how, through relatively minor changes, synthetic biology approaches might be applied to the development of *C. reinhardtii* as an IB platform, and also to enhance the microalgal research field more broadly. It is worth mentioning that other microalgae are being explored for similar applications, and we direct the reader to excellent reviews on the subject (Wijffels *et al*., 2013; Bellou *et al*., 2014; Klok *et al*., 2014; Chauton *et al*., 2015). In addition, several cyanobacterial species are being used for metabolic engineering, including for biofuels (Quintana *et al*., 2011; Berla *et al*., 2013; Wijffels *et al*., 2013). As prokaryotes they are not algae, but their simpler genetic system and ease of transformation can offer advantages. Conversely, they are less versatile than algae in terms of metabolic pathways.

## Molecular Resources for *C. reinhardtii*

**Figure 1:**
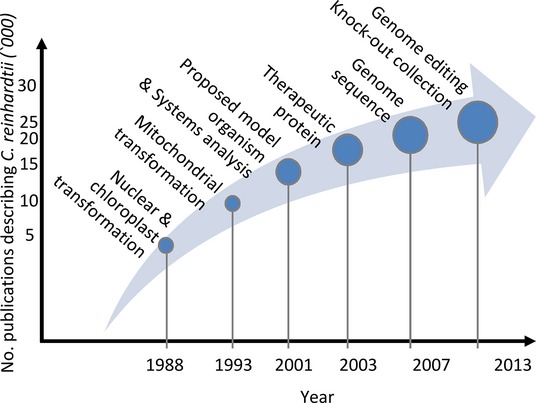
The rise of *C. reinhardtii* as a model system for molecular biology.Major breakthroughs include first nuclear and chloroplast transformations (Boynton *et al*., 1988; Blowers *et al*., 1989; Kindle *et al*., 1989), mitochondrial transformation (Randolph-Anderson *et al*., 1993), systems analysis by proteomics (Hippler *et al*., 2001), the proposal of *C. reinhardtii* as a model organism (Harris, 2001), the production of the first therapeutic recombinant protein in the chloroplast (Mayfield *et al*., 2003), the sequencing of the nuclear genome (Merchant *et al*., 2007) and the publication of genome editing techniques (Sizova *et al*., 2013; Gao *et al*., 2014; Jiang *et al*., 2014) and the creation of a knock-out collection (Zhang *et al*., 2014).

*Chlamydomonas reinhardtii* (Chlorophyta) is a photosynthetic biflagellate microalga that has been studied for >30 years as a model for basic and applied physiology and biochemistry (see articles in the rest of this issue). The number of resources available for the manipulation of *C. reinhardtii* has increased markedly over this period (Figure1). These include well defined protocols for growth, sexual propagation, and mutagenesis, as well as numerous published biochemical, analytical and reporter assays. Supporting research into various aspects of cell biology is the *Chlamydomonas* stock centre, a collection of >300 plasmids and >2700 strains (http://www.chlamy.org). Sequences of the nuclear, chloroplast and mitochondrial genomes have been completed (Merchant *et al*., 2007). The approximately 17 000 genes are well annotated, due to the substantial amount of expression data available, as well as the efforts of the community. The current nuclear genome database, version 5.5, has had most gaps filled and is 93% complete (Blaby *et al*., 2014). This knowledge base is made accessible by web based tools including JGI Phytozome genome browser (Goodstein *et al*., 2012), the functional genomic ChlamyCyc portal (May *et al*., 2009), KEGG metabolic pathway portal (Kanehisa, 2000), and the PlantGDB comparative genomics resource (Duvick *et al*., 2008). The complexity of cellular metabolism is made accessible *in silico* by the generation of genome-scale metabolic models (GSMMs). GSMMs present a snapshot of metabolism in a network format and allow the topology of system to be investigated. Several GSMMs for *C. reinhardtii* have been generated (Boyle and Morgan, 2009; Christian *et al*., 2009; Manichaikul *et al*., 2009; Chang *et al*., 2011; Cogne *et al*., 2011; Dal'Molin *et al*., 2011; Kliphuis *et al*., 2012), and the efficacy of such models has been reviewed recently (Reijnders *et al*., 2014).

Protocols for transformation of the nucleus, chloroplast and mitochondria are available using biolistics (Debuchy *et al*., 1989), electroporation (Shimogawara *et al*., 1998), *Agrobacterium* (Kumar *et al*., 2004) or even simply vortexing with glass beads (Kindle, 1990). Facilitating nuclear transformation is the availability of several selectable markers, including auxotrophic markers, such as *ARG7*, which restores arginine prototrophy (Debuchy *et al*., 1989; Purton and Rochaix, 1995), as well as resistance genes, *BLE* (resistance against phleomycin; Stevens *et al*., 1996), *APHVIII* (paromomycin; Sizova *et al*., 1996), and HYG (hygromycin B; Berthold *et al*., 2002). Transformation of the chloroplast genome can be by homologous recombination, enabling targeted and specific manipulation, although because of the multiple copies of the chloroplast DNA, several rounds of selection are required to obtain homoplasmy (Day and Goldschmidt-Clermont, 2011). Nuclear transformation, on the other hand, is by random integration, and this means that multiple transformants must be screened to obtain stable lines expressing the transgene at the desired level (Debuchy *et al*., 1989). Important recent developments include the generation of a knock-out library consisting of >1600 single gene knockouts (Zhang *et al*., 2014) and the description of two strains, generated by random mutagenesis, which demonstrate enhanced protein expression due to an unknown mechanism that reduces the effects of transgene silencing (Neupert *et al*., 2009).

Complementing these resources are genomics studies of *C. reinhardtii*, of which there an increasing number (Table1), including several aimed at metabolic engineering. Matthew *et al*. (2009) analysed the metabolome of *C. reinhardtii* following sulfur depletion. This analysis demonstrated that between 0 and 24 h post sulfur deprivation, *C. reinhardtii* rapidly metabolised extra-cellular fixed carbon and synthesised the storage compounds starch and lipids in the form of triacylglycerides (TAGs). Over the next 96 h hydrogen production was achieved, peaking at 48 h post sulfur deprivation, while starch reserves were metabolised. However, amino acid and fatty-acid biosynthetic capacity and cellular stores remained intact, suggesting that the observed decline in hydrogen production was not due to metabolic depletion but instead because of the toxic accumulation of fermentation products, formate and ethanol. More recently, Lv *et al*. (2013) analysed the transcriptome of *C. reinhardtii* under exponential growth and lipid accumulation, identifying over 2500 genes that were upregulated. Of these 80% were assigned to functional categories which, as well as lipid biosynthesis, included several central metabolic pathways, suggesting an increase in general metabolism in response to the imposed stress. In addition, 41 transcription factors were differentially expressed under tested conditions. Building upon the genomic analyses are several GSMMs (Reijnders *et al*., 2014), which facilitate predictive attempts to harness the biosynthetic capacity of *C. reinhardtii*. Combined with a legacy of fundamental research and the current impetus placed on microalgae as a potentially sustainable source of energy, these modelling and other systems biology approaches are leading to an enormous increase in our understanding of *C. reinhardtii*, and efforts to manipulate it. At the same time, there has been a rise in the number of other microalgae with sequenced genomes and genomics tools (Figure S1 and Table S1); these data are leading to a much wider knowledge base of these organisms generally.

**Table 1:** Examples of genomic studies completed for the analysis of specific physiological and biochemical characteristics of *C. reinhardtii*

Study	Studies	Reference
Microarrays	Tandem repeat discovery	Zhao *et al*. (2014a)
	Sexual production	Ning *et al*. (2013)
Transcriptomics	Transcription factors	Riaño-Pachón *et al*. (2008)
	Alternative splicing	Labadorf *et al*. (2010)
	Polyadenylation	Zhao *et al*. (2014b)
	Growth adaptation based on natural selection, serial dilution	Perrineau *et al*. (2014)
	TAG production improvement	Goodenough *et al*. (2014)
	Flagella regeneration	Stolc *et al*. (2005)
	CO_2_ effect	Fang *et al*. (2012)
Proteomics	Basal body	Keller *et al*. (2005)
	Mitochondria	van Lis *et al*. (2003)
	Thylakoids	Allmer *et al*. (2006)
Metabolomics	ChlamyCyc database	May *et al*. (2009)
	Partitioning oil and starch	Johnson and Alric (2013)
	S-deprived H_2_ production	Matthew *et al*. (2009)

### Advanced tools for manipulation of the *C. reinhardtii* nucleus

Efforts to express genes in the *C. reinhardtii* nucleus have been underpinned by the elucidation of mechanisms to regulate transgene expression. Examples include the use of promoters (Table2) that have been characterised either as constitutive (e.g. *HSP70A/RBCS2*; Schroda *et al*., 2000) or regulated in response to specific stimuli, such as light (*PSAD*; Fischer and Rochaix, 2001), copper (*CYC6*; Quinn and Merchant, 1995), nitrate (*NIT1*; Ohresser *et al*., 1997), and most recently vitamin B_12_ (*METE*; Helliwell *et al*., 2014). RNA-based elements have also been employed to augment transgene expression (Table2). Introns of the *Rubisco Small Subunit 2* (*RBCS2*) have been used extensively since they were first shown to function as enhancer elements in a manner independent of orientation, and they are effective when positioned either upstream or downstream of the promoter (Lumbreras *et al*., 1998). Thiamine pyrophosphate riboswitches have been identified in the thiamine biosynthetic pathway of *C. reinhardtii* which cause alternative splicing on binding the ligand, (Croft *et al*., 2007). The *THI4* riboswitch has subsequently been used to regulate expression of a nuclear encoded protein, NAC2, which in turn is required for chloroplast gene expression (Ramundo *et al*., 2013). Down-regulation of native *C. reinhardtii* genes has taken advantage of RNA interference, where binding of short fragments of RNA (microRNA or miRNA) complementary to specific mRNAs leads to cleavage and thus silencing of the target gene. *C. reinhardtii* encodes all the components of the RNAi pathway (Casas-Mollano *et al*., 2008), and techniques for artificial-micro-RNA (amiRNA) have been developed (Molnar *et al*., 2009; Zhao *et al*., 2009). In one report, combining amiRNA with the *NIT1* promoter resulted in an inducible gene knockdown system (Schmollinger *et al*., 2010).

**Table 2:** Regulatory elements available for *C. reinhardtii* nuclear gene expression. Categorised based upon functional properties, promoters, regulatory elements, functional peptides, colorimetric reporters and highly expressed strains

Functional element	Name	Phytozyme gene ID	Property	Reference
Promoters	*HSP70A*	Cre08.g372100 (196 bp upstream to ATG)	Typically used as constitutive; expression can be enhanced by high light and temperature	Schroda *et al*. (2000)
	*RBCS2 or HSP70A/RBCS2*	Cre02.g120150 (180 bp upstream to ATG)	Strong constitutive (refer to *HSP70A*)	Lumbreras *et al*. (1998)
	*PSAD*	Cre05.g238332 (822 bp upstream to ATG)	Typically employed as strong constitutive; expression maybe enhanced by high light	Fischer and Rochaix (2001)
	*CYC6*	Cre16.g651050 (127 bp upstream to ATG)	Metal (Cu) responsive	Quinn and Merchant (1995)
	*NIT1*	Cre09.g410950 (282 bp upstream to ATG)	Ammonium responsive	Ohresser *et al*. (1997)
	*ATX1*	Cre09.g392467 (532 bp upstream to ATG)	Iron (Fe) responsive	Fei and Deng (2007)
	*CA1*	Cre05.g248400 (194 bp upstream to ATG)	CO_2_ responsive	Villand *et al*. (1997)
	*SQD2*	Cre01.g038550 (75 bp upstream to ATG)	Phosphate (P) responsive	Iwai *et al*. (2014)
	*CAMV 35S*	–	Enhancer and minimal promoter only	Ruecker *et al*. (2008)
	*METE*	Cre03.g180750 (–574 to –89 bp from ATG)	Cobalamin (B_12_) suppression	Helliwell *et al*. (2014)
Regulatory elements	*RBCS2 intron 1*, *2* and *3*	Cre02.g120150	Enhance gene expression as intron in coding region or 5′UTR	Eichler-Stahlberg *et al*. (2009), Lumbreras *et al*. (1998)
	*THI4*	Cre04.g214150 (1414 bp 5′UTR)	5′UTR, thiamine (B_1_) suppression	Croft *et al*. (2007), Moulin *et al*. (2013)
	RBCS2	32 aa	Rubisco small subunit 2 chloroplast transit peptide	León *et al*. (2007)
Functional peptides	FD	32 aa	Ferredoxin chloroplast targeting transit peptide	León *et al*. (2007)
	ARS2	Cre16.g671350 (63 bp 5′ end from ATG)	N-terminal secretion	Eichler-Stahlberg *et al*. (2009)
	2 x simian virus 40 (SV40)	20 aa	N- terminal nuclear target peptide	Rasala and Mayfield (2014)
	Foot-and-mouth disease virus (FMDV) 2A	20 aa	Translational cleavage peptide	Rasala *et al*. (2012)
	*Gaussia* luciferase (*gLuc*)	555 bp	Luciferase assay	Ruecker *et al*. (2008)
Reporter genes	GFP, mCHERRY, EYFP, DsRED, tdTOMATO, VENUS	708–1437 bp	Fluorescent protein	Rasala *et al*. (2013)
	ARS	Cre16.g671400 (3012 bp cDNA)	Chromogenic assay	Davies *et al*. (1992)
Highly expressed host strains	UVM4 and UVM11	–	High expression host, transgene silencing supressed	Neupert *et al*. (2009)

Several different reporter genes are available, both fluorescent and colorimetric, and in most instances these have been modified for expression in *C. reinhardtii*; the unusually high GC content of 65% (Merchant *et al*., 2007) means that most heterologous genes need to be codon optimised before they are expressed efficiently (e.g. Fuhrmann *et al*., 1999). Expressed proteins can be directed to different subcellular locations, or for secretion, using characterised targeting peptides, and tags for immunodetection or purification of expressed proteins have been developed (Eichler-Stahlberg *et al*., 2009). Eukaryotic cleavage peptides, such as viral 2A peptides (Rasala *et al*., 2012), have been demonstrated to work in *C. reinhardtii*, thus in principle allowing expression of two or more proteins from a single transgene construct. This would overcome potential difficulties in the limited number of promoters available, repeated use of which would be likely to increase the problems of gene silencing.

In the last 2 years, reports of advanced techniques for genome editing of *C. reinhardtii* have appeared. The first demonstration of genome editing was via zinc finger nucleases, which work by fusing a DNA binding domain from naturally occurring zinc finger nuclease with a DNA cleavage domain. Sizova *et al*. (2013) transformed *C. reinhardtii* with a heterologous non-functional *APHVIII* gene, interrupted with a 24-bp *COP3* sequence, the target of the zinc finger nuclease. Subsequent transformation with the gene encoding the zinc finger nuclease, expressed from the *HSP70A* promoter, and a 120-bp sequence of DNA complementary to the non-functional region of *APHVIII*, followed by selection for paromomycin resistance, generated several colonies, indicating restoration of *APHVIII* gene function. No spontaneous resistance was apparent in the controls. The methodology was subsequently used to introduce targeted mutations into the native *COP3* gene. The second example of genome editing employed Transcription Activator-Like Effectors (TALE) for specific activation of target genes (Gao *et al*., 2014). In this work, the endogenous arylsulfatase genes, *ARS1* and *ARS2*, were targeted via their promoters. Nuclear expression of the respective TALE, from a *HSP70A/RBCS2* promoter and *RBCS2* terminator cassette increased the expression of each *ARS* at the level of both the transcript and protein, a fact confirmed by an assay of arylsulfatase activity. Finally the efficacy of the Clustered Regularly Interspersed Short Palindromic repeat (CRISPR) system was tested (Jiang *et al*., 2014). CRISPR associated protein 9 (CAS9) is an RNA-guided DNA nuclease that has been employed to introduce targeted mutations into eukaryotic genomes. No stable transformants of *C. reinhardtii* expressing the CAS9 protein could be recovered, suggesting that the protein was toxic (Jiang *et al*., 2014). As a result the authors employed transient expression in an attempt to orchestrate targeted mutagenesis of four independent gene targets, *HYG*, mutant green fluorescent protein (*mGFP*), *Gaussia* luciferase (*gLUC*) and peptidyl-prolyl *cis-trans* isomerase (*FKB12*). A PCR assay was employed to enrich for and detect the presence of Cas9 mediated mutations. Although the system demonstrated low efficiency, with only a single *FKB12* mutant obtained, it provides a benchmark for future research in this area.

In spite of our ability to manipulate the genome of *C. reinhardtii*, few examples of actual metabolic engineering are available in the literature. Of these, engineered expression of native genes for diacylglycerol acyltransferases (DGATs) provides a good example of the current status. In this, three type-2 *DGAT* genes were expressed constitutively with the *PSAD* promoter and 3′UTR, and their impact on TAG biosynthesis and profile analysed (La Russa *et al*., 2012). Although expression varied due to random integration, *DGAT* mRNA levels were increased 1.7- to 29.1-fold compared to controls. In spite of this no significant increase in lipid biosynthesis was observed, possibly due to induction of lipid degradation pathways (Nguyen *et al*., 2011). A second example is provided by the attempt to engineer *C. reinhardtii* to make the keto-carotenoids, astaxanthin and canthaxanthin. Constitutive expression of a β-carotene ketolase gene (*BKT1*), derived from the green microalga *Haematococcus pluvialis*, in the *C. reinhardtii* nucleus using the *RBCS2* promoter, combined with targeting to the chloroplast via RBCS2 or ferredoxin (FD) transit peptides (Table2) resulted in the undesired biosynthesis of 4-keto-lutein (León *et al*., 2007). Possible reasons for this failure include lack of substrate accessibility in the thylakoid membranes where the carotenoids are synthesised, and/or the inability of the heterologous β-ketolase to integrate into the carotenogenic enzyme complexes. For both this, and the example of expression of DGATs, optimisation would require many different genetic constructs to be tested, including down-regulation of endogenous pathways as well as expression of transgenes in the same cell line. The current tools available for manipulation of *C. reinhardtii* limit considerably the ability to carry out these combinatorial studies.

### Engineering the *C. reinhardtii* Chloroplast

From an engineering perspective, the chloroplast provides a unique cellular compartment that harbours numerous essential processes, including those of biotechnological importance such as carotenoid and fatty-acid biosynthesis. The chloroplast genomes of more than 20 species are now accessible to genetic modification (Day and Goldschmidt-Clermont, 2011; Maliga, 2012). However, chloroplast engineering is routine for only three species, *Nicotiana tabacum* (Golds *et al*., 1993), *N. benthamiana* (Davarpanah *et al*., 2009), *C. reinhardtii* (Blowers *et al*., 1989). This further highlights the importance of *C. reinhardtii* as an IB platform. Working with the chloroplast provides a number of unique advantages, for example, the chloroplast genome is derived from a cyanobacterial genome that has been reduced by two orders of magnitude (Scharff and Bock, 2014). Its prokaryotic origin allows genes to be co-ordinated into operons, and to integrate DNA in a targeted manner via homologous recombination, so that mutant phenotypes can be complemented and resistance markers recycled to generate ‘marker-less’ strains (Fischer *et al*., 1996; Bateman and Purton, 2000) (Table3). Moreover, some established bacterial resources also work in the *C. reinhardtii* chloroplast, including the *lac* repressor system (Kato *et al*., 2007). This is useful as it has been reported that native promoters maybe compromised by tight regulation of essential photosynthetic processes. For example, the promoter and 5′UTR of the *psbA* gene, which encodes the D1 subunit of photosystem II, can only be used in a *psbA*-deficient background because of D1-dependent auto-repression (Rasala *et al*., 2011). In spite of this, some combinations of promoter and 5′UTR have been identified that achieve efficient transgene expression in photosynthetic backgrounds, such as the 16S rRNA promoter-*atpA* 5′UTR hybrid (Rasala *et al*., 2011), and there are several reports of expression of single proteins being produced within the *C. reinhardtii* chloroplast (Tissot-Lecuelle *et al*., 2014). However, examples of metabolic engineering are much fewer in number. The most relevant example is that of enhanced bio-hydrogen production. Wu *et al*. (2011) expressed codon optimised *hemH* and *lba* to enhance cellular respiration, reducing the dissolved oxygen concentration in the media, thereby causing an up regulation of hydrogenase genes *hydA1* and *hydA2*. Nonetheless, as with examples in the nucleus, improvements obtained through chloroplast metabolic engineering are limited compared with what has been achieved in other biological platforms.

**Table 3:** Regulatory elements available for the *C. reinhardtii* chloroplast. Chloroplast functional parts are classified as transcriptional leaders (promoter and 5′UTR), chloroplast-targeted peptides, selectable markers and destination sequences for homologous recombination

Functional element	Name	Properties	Source
Transcriptional leaders	*AtpA*, *psbD*	Constitutive expression	Barnes *et al*. (2005)
	*psaA-exon1*	Strong expression	Michelet *et al*. (2011)
Chloroplast-targeted peptides	*psaD*	35 aa N-terminal	Fischer and Rochaix (2001)
	*rbcS2*, *FD*	32 aa N-terminal	León *et al*. (2007)
Selectable marker	*rrnS* and *rrnL point mutations*	Spectomycin and erythromycin resistance	Kindle *et al*. (1991)
	*arg9*	Arginine complementation	Remacle *et al*. (2009)
	*aadA*	Spectinomycin resistance	Goldschmidt-Clermont (1991)
	*aphA6*	Resistance kanamycin	Bateman and Purton (2000)
	*GFP*	Fluorescent reporter	Franklin *et al*. (2002)
	*gusA*	β-Glucuronidase activity	Sakamoto *et al*. (1993)
	*atpB*	5-Fluorodeoxyuridine resistance	Kindle *et al*. (1991)
	*coda*	Cytosine deaminase sensitivity to 5-fluorocytosine	Young and Purton (2014)
Destination sequences for homologous recombination	*p322*	*psbA* and 16S rRNA	Manuell *et al*. (2007), Barnes *et al*. (2005)
	*patpint-cg11 (atpB-int)*	Inverted repeat and *atpB* 3′UTR	Nickelsen *et al*. (1994)
	*pLM7 (IR-int)*	*psbA* and 5S/23S	Michelet *et al*. (2011)
	*p72B*	*psbH* and *psbN*	Bateman and Purton (2000)
	*p71*	*tscA* and inverted repeat	Kindle *et al*. (1991)

## Harnessing Biological Systems for Technology

Industrial biotechnology employs biological systems, mainly microbes, for the production of commodities. Today these range from fuels, to platform chemicals, and many high-value products, including a vast range of therapeutics and pharmaceuticals. IB has its origins in fermentation practices for beer, wine and breadmaking, but as microbiology emerged as a science, the ability to control these technologies proceeded in parallel, and led to many important compounds being made this way (reviewed in Buchholz and Collins, 2013). A noteworthy example is penicillin from the fungus *Penicillium notatum*, as well as several other naturally occurring antibiotics from soil bacteria. The advent of genetic manipulation enabled the optimisation of biological production systems and enormously expanded the portfolio of compounds that could be produced by IB, including the ability to produce human insulin and growth hormone as recombinant proteins, avoiding the side effects of use of animal forms of the hormones (Vajo *et al*., 2001). Today, as well as pharmaceuticals, products from IB are an everyday feature of life, from vegetarian cheeses made with recombinant rennet, to plastics made from platform chemicals, to enzymes in washing powders (Goel *et al*., 2012).

**Figure 2:**
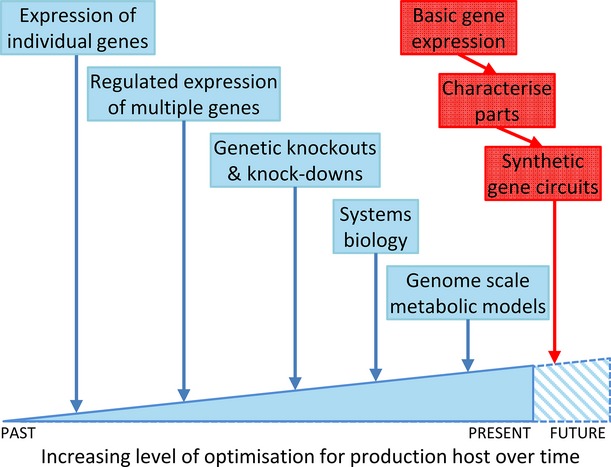
Schematic of host optimisation via metabolic engineering and synthetic biology.The chronologic development of a biological host via metabolic engineering (blue), including the recent advent of systems biology and genome-scale metabolic models. Synthetic biology (red) has the potential to expedite this process in new biological systems, including microalgae, providing an opportunity to proceed directly from basic knowledge and capacity to the generation of highly optimised productive hosts.

The best known IB platforms are *E. coli* and *S. cerevisiae*, their utility being derived from decades of research that has progressed through various stages, ranging from the introduction of individual genes using standard expression systems, to the regulation of complex biochemical pathways using highly regulated genetic circuits (Figure2). Parallel to the development of tools for manipulation, the application of systems biology and GSMMs have enabled rational modification of discrete pathways (Yu *et al*., 2011; Chen and Zeng, 2013), whilst improvement of platform strains with respect to growth and productivity within the fermenter, as well as downstream processing, has led to optimised hosts for different bioprocesses (Goel *et al*., 2012). This progression is demonstrated by the work on the optimisation of *E. coli* strains expressing a gene encoding amorphodiene synthase from *Artemisia annua*, which catalyses the conversion of the isoprenoid intermediate farnesyl pyrophosphate into amorphodiene. Starting with a landmark metabolic engineering paper, Martin *et al*. (2003) introduced a yeast mevalonate pathway into *E. coli* to enhance production of the isoprenoid precursor, isopentenyl pyrophosphate. This increased amorphodiene biosynthesis 36-fold, to 24 μg mL^−1^. This strain was employed in several subsequent studies to achieve iterative improvements in target compound production by elucidating metabolic limitations in precursor biosynthesis and improving carbon flux towards the end products. First, expression of the mevalonate pathway gene 3-hydroxy-3-methyl-glutaryl-coenzyme A (HMG-CoA) reductase was modulated to improve precursor biosynthesis leading to a three-fold (ca. 70 μg mL^−1^) increase over the original strain (Pitera *et al*., 2007). Following this, mevalonate kinase and amorphadiene synthase were identified as rate limiting, and optimisation of promoter strength and gene copy number increased productivity seven-fold (ca. 160 μg mL^−1^) over the original construct (Anthony *et al*., 2009). This moved the metabolic bottleneck back to HMG-CoA reductase, and the introduction and optimisation of an alternative HMG-CoA reductase, derived from *Delftia acidovorans*, led to levels of 700 μg mL^−1^ amorphodiene (Ma *et al*., 2011). Most recently, Dahl *et al*. (2013) identified farnesyl pyrophosphate as a toxic intermediate and introduced dynamic control to regulate its synthesis and consumption. The application of systems biology methods identified promoters induced in response to intermediate accumulation, and these were employed to drive the expression of both the farnesyl pyrophosphate biosynthetic pathway and the amorphadiene biosynthetic genes, resulting in a doubling of the amorphadiene titre, to 1.6 mg mL^−1^. This approach is now routine in IB where iterative improvements enable optimisation of inputs and gene expression to maximise the economic viability of a bioprocess.

Tangential to these developments has been the rise of synthetic biology. Much of the current focus of this discipline is on the development and analysis of genetic circuits, emergent behaviour and minimal genomes (Juhas *et al*., 2011), as well as the generation of non-natural biological systems with an expanded genetic code (Neumann and Neumann-Staubitz, 2010). Nonetheless, the potential for synthetic biology to contribute to IB is significant (Khalil and Collins, 2010; Yadav *et al*., 2012).

## Synthetic Biology Allows Rational Design of Biological Systems

**Figure 3:**
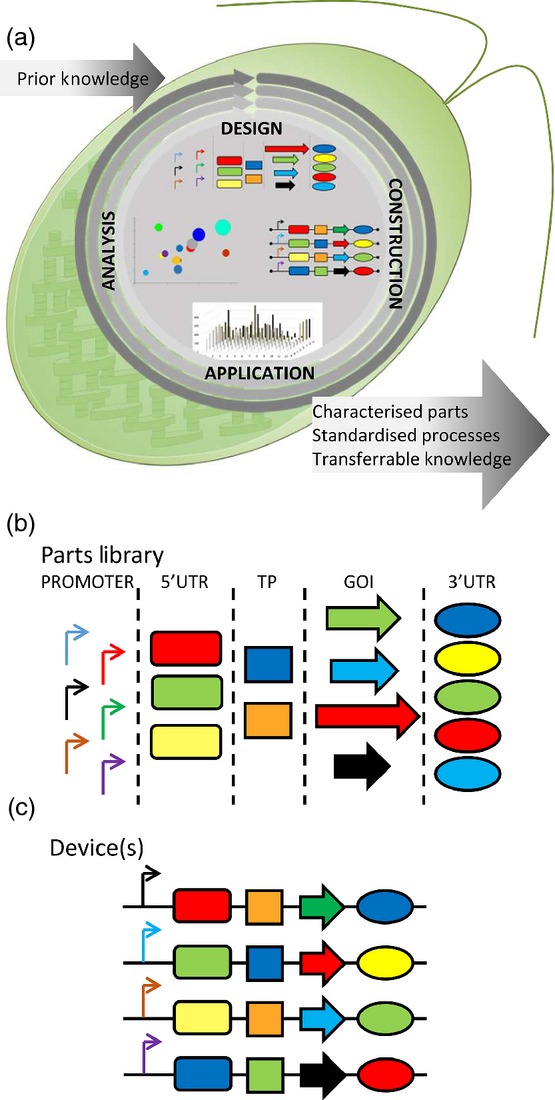
Schematic representation of a synthetic biology workflow.An example investigation may aim to increase metabolite *x* through heterologous expression of gene *y*. Prior to this knowledge must be generated to describe the function of individual parts, promoters, 5′UTRs, transit peptides (TP), (trans)genes of interest (GOI) and 3′UTRs, and/or introns to enhance mRNA stability. Achieved through progress from design through construction application and analysis (including *in silico* models), when completed in a standardised manner this represents a single iteration of the cyclic process (a). The completion of several rounds of characterisation generates the knowledge required assign functional parameters to individual (generic) parts. These parts can be considered in a discrete manner (b) providing the opportunity to deconstruction of complex modules and recombine the parts a rational manner to generate novel functional devices (c) that have a predictable output.

Synthetic biology draws on principles first employed in the electronics field, and aims to create or (re)design complex biological circuits, networks or whole organisms in a rational way, combining computational models with assembly of standard or modularised components, to create useful outputs. Synthetic biology is characterised by an iterative workflow of continuous improvement, with generated knowledge informing subsequent rounds of design (shown schematically in Figure3a). The basis is the use of standard *parts* (Figure3b) for the design and engineering of novel biological systems, in this example DNA sequences such as promoters, terminators or introns. These parts or pathways may be described as *orthogonal*, that is they are transferrable between different cellular hosts with little/no impact on function (i.e. platform independent). They may be *insulated*, so they are unaffected by host cell metabolism, while an approach may be said to lead to *abstraction*, allowing design at higher levels of complexity because work builds upon well characterised inputs. This makes knowledge transferable, as a combination of elements that lead to a predetermined expression level of a transgene could be employed in subsequent studies to drive the expression of other transgenes. The aim is that, once sufficiently well characterised, the design process no longer needs to consider individual DNA parts. Instead these may be combined into expression cassettes or *devices* (Figure3c) with novel but predictable outputs. This will allow the field to progress more efficiently by building directly upon work that preceded it. Table4 provides a summary of other commonly used synthetic biology terms.

**Table 4:** Summary of common synthetic biology terms

Keyword	Description
Abstraction	Through standardisation of individual parts these may be combined into simple devices with predictable outputs, combination of these devices allows design at a higher level of complexity, i.e. circuits or systems. This is the concept of abstraction
Boolean logic gates	An engineering principle in which an output is dependent on one or more specific inputs, and where the output is 1 or 0, true or false, on or off
Chassis	The framework on which to base the standardised parts. Effectively, a host organism into which standard genetic constructs can be introduced easily
Device	A DNA construct incorporating required part(s) for desired expression of a transgene
Modularity	A design concept in which parts are considered as discrete elements. Characterised parts can be combined to create an expression cassette which, due to its predictable function, is considered a modular element in its own right, becoming a part
*In-silico* modelling	The application of informed metabolic models to inform experimental design and allow hypothesis-driven research into complex systems
Orthogonal	The function of a DNA part independently of: (i) cellular platform; and/or (ii) cellular context
Part(s)	DNA sequence(s) that encode a biological function, e.g. promoter, intron, 3′UTR, reporter gene
Standardisation	The application of uniformed strategies to assess, characterise or validate biological parts or processes. Examples include assembly protocols, reporter assays, or selection methods

### Synthetic biology in action – *Escherichia coli*

*Escherichia coli* is the best understood and most advanced biological platform available, demonstrating great utility for the development of ground-breaking molecular, genetic and biochemical techniques. As such it has been widely employed to pioneer synthetic biology (Cameron *et al*., 2014). For example, there is now a Registry of Standard Biological Parts, or Biobricks (Knight, 2003), the number of which now exceeds 12 000 (Vilanova and Porcar, 2014). Public access allows researchers to explore novel functionalities and applications, in return for contributing to the centralised knowledge database. This foundation provides the ability to engineer strains and create genetic circuits, such as the use of a two-component sensor and response system where genes were regulated in response to different light wavelengths (Olson *et al*., 2014). The system was composed of a histidine kinase with an N-terminal phytochrome domain sensor and a C-terminal bifunctional kinase-phosphatase signalling domain. Using biological information and modelling, the system was manipulated to regulate protein expression in response to different wavelengths and pulse profiles of light using an automated illumination program. The binary states demonstrated by this system provide a basis for future logic based gene circuits.

*Escherichia coli* synthetic biology has already advanced to population-scale regulation using synthetic networks. An example of this is the synchronisation of genetic clocks by post-translational events. Prindle *et al*. (2014) employed intracellular negative-feedback and a quorum-sensing oscillator in combination with proteins tagged for rapid protease catalysed degradation via the CLPXP signal. By coupling these processes the authors engineered a system in which the response delay was reduced by an order of magnitude over systems coupled at the level of transcription. Modification of the linker sequence length between the peptide and the degradation tag made the timing of the response tunable, offering the potential to create optimised expression profiles for synthetic gene circuits. As an illustration, a useful application of this for metabolic engineering would be regulation of key mevalonate biosynthesis genes in the amorphodiene strain discussed above.

### Synthetic biology in action – *Saccharomyces cerevisiae*

The major chassis in eukaryotes is *S. cerevisiae*. This unicellular yeast benefits from numerous community resources that support fundamental research, including knock-out collections in which each protein-coding region has been systematically disrupted both individually (Dujon, 1998; Winzeler *et al*., 1999), and in pairs (Tong *et al*., 2001), allowing investigation of lethality and gene relationships. Aspects of *S. cerevisiae* research have moved to adopt the principles of synthetic biology. A pioneering example of this is the creation of an entirely artificial yeast chromosome (Annaluru *et al*., 2014), engineered to streamline and condense the encoded genetic information and allow re-organisation of coding regions on a massive scale. This paves the way for rapid laboratory-based evolution to generate novel phenotypes with biotechnological relevance. Further examples of how synthetic biology has been applied to *S. cerevisiae* include the development of novel synthetic inducible promoters, regulated by a ligand that does not influence cell physiology (i.e. is insulated), which are also tunable. McIsaac *et al*. (2014) constitutively expressed an artificial transcription factor (ATF) in a cell line harbouring a green fluorescent protein (GFP) reporter that was regulated via a synthetic promoter containing one or several binding sites for the ATF. The reporter was expressed in the presence of the inducer, β-estradiol, and modification to the number and position of the ATF binding sites provided tunable expression upon induction, with a dynamic range that spanned three orders of magnitude.

In another study, Yofe *et al*. (2014) investigated the ability of non-coding sequences to regulate gene expression in *S. cerevisiae*. Creating a library of 240 reporter lines, each containing a different intron within the reporter gene coding sequence, the authors observed variation in expression spanning 100-fold. These lines demonstrated robustness in expression level and a capacity for tunable gene expression, achieved through the modification of key intron motifs and/or the folding energy of the intron-exon boundary. Finally, to allow rational design, the authors developed a computational model that fitted the experimental performance of tested sequences.

To demonstrate the power of a combinatorial approach, the five genes of the uncharacterised violacein biosynthetic pathways were targeted. In this, five promoters which spanned nearly three orders of magnitude in expression level were employed to drive expression of the violacein biosynthetic genes from BBa_K274002, a plasmid from the Registry of Standard Biological Parts (Knight, 2003). A rapid PCR genotyping method combined with computational modelling to integrate experimental data enabled the prediction of pathway compositions that would minimise intermediate accumulation and/or maximise violacein biosynthesis. In doing so the authors demonstrated pathway optimisation without prior knowledge of absolute protein or metabolite levels, enzyme kinetics or thermodynamics, or even the order of the reactions (Lee *et al*., 2013).

## Development of a *C. reinhardtii* Industrial Biotechnology Platform

The approaches presented above provide an overview of what is currently possible with well characterised IB platforms, and how a synthetic biology approach would augment both the range and amount of compounds that could be produced, and the rate of progress. For *C. reinhardtii*, although our ability to manipulate it genetically is advancing rapidly, many limitations remain for effective metabolic engineering. For example, fewer than 100 protein-coding genes from the *C. reinhardtii* genome have been functionally validated, compared with the approximately 6800 genes that have been characterised experimentally in *Arabidopsis thaliana* (Koornneef *et al*., 2004). Additionally, the number of tools to regulate transgene expression (Tables2 and 3) is small compared with standard IB hosts. There are also the well known complications of integration of transgenes into the nucleus via non-homologous end joining, and extensive gene silencing. A significant additional challenge is the fact that regulatory networks at the genetic, cellular, and metabolic levels in *C. reinhardtii* are almost completely unexplored, so that attempts at manipulation will be effectively empirical, whereas there are many studies of the interactome of *E. coli* (Juan *et al*., 2008) and *S. cerevisiae* (Ito *et al*., 2001). Although undoubtedly some of these knowledge gaps for *C. reinhardtii* will be filled in the near future (as discussed above), it should be noted that no biological chassis evolved to be optimal for scientific research, with extensive engineering required to create today's *E. coli* strains for routine protein expression (Goel *et al*., 2012; Buchholz and Collins, 2013). Thus rather than waiting for this to be true for *C. reinhardtii*, would it be possible to expedite progress by applying current leading concepts, techniques and technologies from synthetic biology? This might lead to quicker solutions to current research questions and, importantly, provide the opportunity to develop *C. reinhardtii* production systems that exceed the capacity of current metabolic engineering approaches. Adopting standardisation, modularity, and design/testing does not require drastic changes in approach, nor does it render all prior knowledge obsolete. Instead this progression can be achieved by subtle changes to minimise experimental variables and standardise workflows, creating a cycle of perpetual improvement.

**Figure 4:**
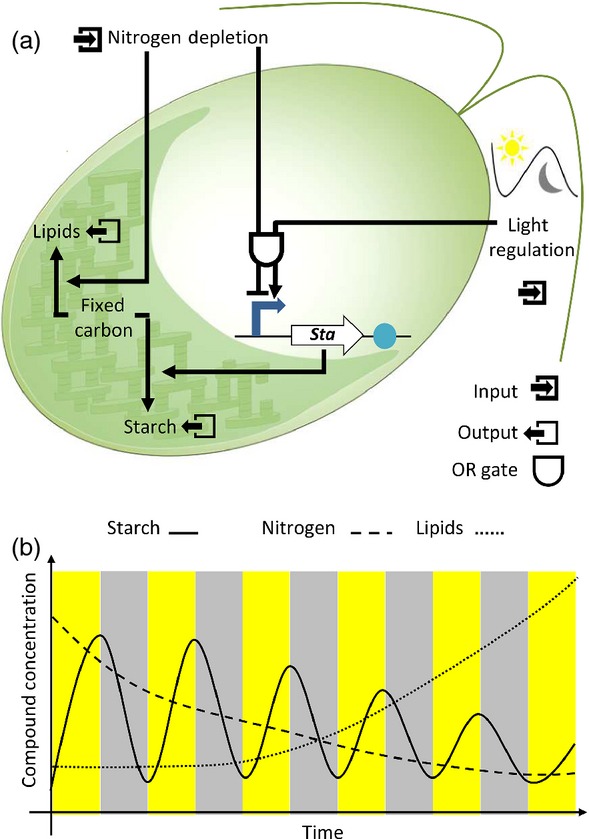
A schematic of synthetic biology applied to create a circuit for enhanced lipid production.(a) A representation of a synthetic gene circuit in a starchless mutant cell line. Starch biosynthesis is complemented by the *STA* gene, in a light-regulated manner (Light; input). This regulation is overruled under nitrogen stress (nitrogen; input) through an OR logic gate. Down-regulation of starch biosynthesis coincides with the induction of native TAG biosynthetic pathways, providing a larger substrate pool for increased TAG production.(b) A schematic model employed to correlate the relationship of starch, nitrogen and lipids with the inputs of light and cellular nitrogen concentration.

To demonstrate this, let us consider an example of improved TAG biosynthesis for biodiesel production (Figure4). When cultured under diurnal conditions, i.e. with a light/dark photoperiod, *C. reinhardtii* will photosynthesise during the light, fixing carbon dioxide and storing any excess as starch. In the dark the accumulated starch is consumed to support cellular activity. When exposed to nutrient stress, such as nitrogen depletion, *C. reinhardtii* accumulates much higher amounts of storage molecules, principally starch and TAG (Merchant *et al*., 2012). Under conditions where the desired product is TAG, starch biosynthesis may be considered an undesired carbon sink. This principle was demonstrated through the analysis of mutants deficient in starch biosynthesis (Wang *et al*., 2009). However, starchless mutants are not able to be propagated under diurnal conditions because they lack the required starch to survive periods of darkness (Davey *et al*., 2014). To overcome this and optimise TAG production one may therefore consider a synthetic circuit that links nitrogen depletion and starch biosynthesis in a manner that would enhance carbon flux into TAG production only under nitrogen stress (shown schematically in Figure4a). Employing the workflow presented in Figure3, a regulatory system could be identified and used to regulate the expression of a starch biosynthesis gene in response to light or nitrogen depletion. Once suitable elements are identified data describing expression characteristics could be integrated into an *in silico* model to generate predicted outputs (Figure4b), and then tested to find the optimal combination of parts for the device. The next stage would be to engineer the system such that TAG production was induced independently of nitrogen depletion. Complementation of a starchless phenotype using this regulatory system would restore starch biosynthesis to that of a wild-type strain, with expression induced in response to light availability (input; light). Because the same system would be down-regulated in response to nitrogen stress (input; nitrogen depletion), this would over-ride the light-based regulation (OR gate logic). The designed circuit would, when exposed to nitrogen stress, suppress starch biosynthesis as native TAG production pathways are induced, producing more TAG because of increased fixed carbon availability.

## Conclusion

Microalgae are of interest to academic and commercial stakeholders due to their unique biology, which encompasses facets of bacterial and yeast systems, and their potential as a biotechnology platform. Although the microalgal research field is less well developed than for the former organisms, the model green alga, *C. reinhardtii*, is positioned for development as an IB platform. It is evident that the major limitation now lies in our capacity to engineer *C. reinhardtii* efficiently. By taking a synthetic biology approach, progress within the field should be accelerated.

One final point to make is that the diversity of microalgae means that *C. reinhardtii* does not provide all of the attributes assigned to ‘microalgae’. That said, its development as an IB platform would undoubtedly benefit the entire microalgal community, and pave the way for subsequent more efficient development of alternative microalgal hosts of biotechnological importance, including *P. tricornutum*, *Thalassiosira pseudonana*, *O. tauri* and *Nannochloropsis* spp. (Table S1). These will offer a wider range of products, and possibly other advantages such as homologous recombination, which has been reported for the latter two species. It is important to acknowledge that although *E. coli* and *S. cerevisiae* are the best known IB platforms, many other bacteria and yeasts are employed in million dollar biotechnology sectors for the production of value-added products. The time is right to consider extending this to microalgae to address the desire to improve the sustainability of these processes (Langevels *et al*., 2010).

## Supporting Information

Figure S1 Algal-biotechnological advancements from a phylogenetic perspective.

Table S1 Overview of microalgae that are transformable and/or have a published genome
