# Supplementary figures and images for: Establishing Chlamydomonas reinhardtii as an industrial biotechnology host
Source: Plant J. 2015 Mar 8;82(3):532–46. doi: 10.1111/tpj.12781 (PMC4515103; doi:10.1111/tpj.12781)

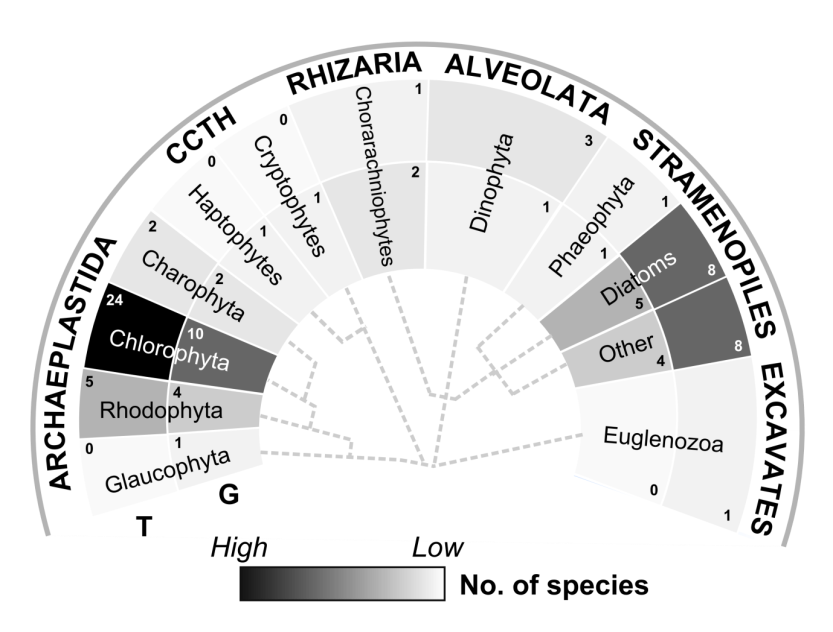

Supplement: Figure S1 — Algal-biotechnological advancements from a phylogenetic perspective. [file tpj0082-0532-sd1.tif]
