# Supplementary material for: Establishing Chlamydomonas reinhardtii as an industrial biotechnology host
Source: Plant J. 2015 Mar 8;82(3):532–46. doi: 10.1111/tpj.12781 (PMC4515103; doi:10.1111/tpj.12781)
Supplement: Table S1 — Overview of microalgae that are transformable and/or have a published genome [file tpj0082-0532-sd2.docx]

**Table S1**. Overview of microalgae that are transformable and/or have a published genome.

| **Genus** | **Species** | **Strain** | **Phylogenetic Grouping** | **Transformable**  **(N: nuclear; C: chloroplast) genome?** | **Sequenced Genome?** | **Reference** |
| --- | --- | --- | --- | --- | --- | --- |
| *Cyanophora* | *paradoxa* | CCMP329 | Glaucophyta | - | ✓ | (Price *et al.*, 2012) |
| *Cyanidioschyzon* | *merolae* | 10D | Rhodophyta | ✓ (N)* | ✓ | (Minoda *et al.*, 2004; Matsuzaki *et al.*, 2004) |
| *Galdieria* | *sulphuraria* | 074W | Rhodophyta | - | ✓ | (Barbier *et al*. 2005) |
| *Gracilaria* | *changii* | - | Rhodophyta | ✓ (N) | - | (Gan *et al.*, 2003) |
| *Kappaphycus* | *alvarezii* | - | Rhodophyta | ✓ (N) | - | (Wang *et al.*, 2010; Kurtzmann and Cheney, 1991) |
| *Porphyridium* | sp. | UTEX 637 | Rhodophyta | ✓ (N, C) | - | (Lapidot *et al.*, 2002) |
| *Porphyridium* | *purpureum* | CCMP 1328 | Rhodophyta | - | ✓ | (Bhattacharya *et al.*, 2013) |
| *Porphyra* | *yezoensis* | U-51 | Rhodophyta | ✓ (N) | ✓ | - (Liu *et al.*, 2003; Mei *et al.*, 1998; Nakamura *et al.*, 2013) |
| *Closterium* | *peracerosum-strigosum-littorale* | NIES-67 (mt^+^) and NIES-68 (mt^−^) | Charophyta | ✓ (N) | - | (Abe *et al.*, 2011) |
| *Klebsormidium* | *flaccidum* | NIES-2285 | Charophyte | - | ✓ | (Hori *et al.*, 2014) |
| *Penium* | *margaritaceum* | - | Charophyte | ✓ (N) | ✓ | (Sørensen *et al.*, 2014) |
| *Botryococcus* | *braunii* | - | Chlorophyta | - | ✓(pending, JGI) | - |
| *Bathycoccus* | *prasinos* | RCC1105 | Chlorophyta | - | ✓ | (Moreau *et al.*, 2012) |
| *Chlamydomonas* | *reinhardtii* | CC-503 *cw92 mt*+ (genome) | Chlorophyta | ✓ (N, C) | ✓ | (Boynton *et al*., 1988; Kindle, 1989; Merchant *et al.*, 2007) |
| *Chlorella* | *ellipsoidea* | - | Chlorophyta | ✓ (N) | - | (Chen *et al.*, 2001; Liu *et al.*, 2013) |
| *Chlorella* | *Kessleri* | 211-11H | Chlorophyta | ✓ (N) | - | (El-Sheekh, 1999) |
| *Chlorella* | *minutissima* | *UTEX 2219* | Chlorophyta | ✓ (N) | - | (Hsieh *et al.*, 2012) |
| *Chlorella* | protothecoides | sp. 0710 | Chlorophyta | - | ✓ | (Gao *et al.*, 2014) |
| *Chlorella* | *Saccharophila* | 211-a | Chlorophyta | ✓ (N) | - | (Maruyama *et al.*, 1994) |
| *Chlorella* | *sorokiniana* | - | Chlorophyta | ✓ (N) | - | (Dawson *et al.*, 1997) |
| *Chlorella* | variabilis | NC64A | Chlorophyta | - | ✓ | (Blanc *et al.*, 2010) |
| *Chlorella* | *vulgaris* | UMT-M1 | Chlorophyta | ✓ (N) | - | (Chow and Tung, 1999; Niu *et al.*, 2011; Cha *et al.*, 2012) |
| *Chlorella* | zofingiensis | ATCC-30412 | Chlorophyta | ✓ (N) | - | (Liu *et al.*, 2014) |
| *Dunaliella* | *bardawil* | V-101 | Chlorophyta | ✓ (N) | - | (Anila *et al.*, 2011) |
| *Dunaliella* | *salina* | UTEX-1644 | Chlorophyta | ✓ (N) | - | (Geng *et al.*, 2003; Tan *et al.*, 2005; Feng *et al.*, 2009) |
| *Dunaliella* | *viridis* | - | Chlorophyta | ✓ (N) | - | (Sun *et al.*, 2006) |
| *Eudorina* | *elegans* | UTEX 1193 | Chlorophyta | ✓ (N) | - | (Lerche and Hallmann, 2013) |
| *Gonium* | *pectorale* | SAG 12.85 | Chlorophyta | ✓ (N) | - | (Lerche and Hallmann, 2009) |
| *Haematococcus* | pluvialis | SAG 19-a and Flotow NIES-144 | Chlorophyta | ✓ (N, C) | - | (Teng *et al.*, 2002; Kathiresan *et al.*, 2009; Saei *et al.*, 2012; Gutiérrez *et al*., 2012) |
| Helicosporidium | sp. | ATCC50920 | Chlorophyta | - | ✓ | (Pombert *et al.*, 2014) |
| *Lobosphaera* | *(Parietochloris) incisa* | SAG 2468 | Chlorophyta | ✓ (N) | - | (Zorin *et al.*, 2014) |
| *Micrasterias* | *denticulata Breb.* | - | Chlorophyta | ✓ (N) | **-** | (Vannerum *et al.*, 2010) |
| *Micromonas* | *pusilla* | CCMP1545 and RCC299 | Chlorophyta | - | ✓ | (Worden *et al.*, 2009) |
| *Ostreococcus* | *lucimarinus* | CCMP2514 | Chlorophyta | - | ✓ | (Palenik *et al.*, 2007) |
| *Ostreococcus* | *tauri* | OTH95 | Chlorophyta | ✓ (N)* | ✓ | (Derelle *et al.*, 2006; Corellou *et al.*, 2009; van Ooijen *et al.*, 2012; Lozano *et al*., 2014) |
| *Pandorina* | *morum* | SAG 32.96 | Chlorophyta | ✓ (N) | **-** | (Lerche and Hallmann, 2014) |
| *Parachlorella* | *kessleri* | - | Chlorophyta | ✓ (N) | **-** | (Rathod *et al.*, 2013) |
| *Platymonas (Tetraselmis)* | *subcordiformis* | - | Chlorophyta | ✓ (N, C) | **-** | (Cui *et al.*, 2011; Cui *et al.*, 2014) |
| *Pseudochoricystis* | *ellipsoidea* | - | Chlorophyta | ✓ (N) | **-** | (Imamura *et al.*, 2012) |
| *Scenedesmus* | *obliquus* | FSP-3 | Chlorophyta | ✓ (N) | **-** | (Guo *et al.*, 2013) |
| *Ulya* | *Lactuca L.* | - | Chlorophyta | ✓ (N) | - | (Huang *et al.*, 1996) |
| *Volvox* | *carteri* | nagariensis | Chlorophyta | ✓ (N)* | ✓ | (Schiedlmeier *et al.*, 1994; Prochnik *et al.*, 2010; Hallmann *et al.,* 1997) |
| *Emiliania* | *huxleyi* | CCMP 1516 | Haptophyta | **-** | ✓ | (Read *et al.*, 2013) |
| *Guillardia* | *Theta* | CCMP2712 | Cryptomonad | - | ✓ | (Curtis *et al.*, 2012) |
| *Cyclotella* | *cryptica* |  | Diatom | ✓ (N) | - | (Dunahay *et al.*, 1995) |
| *Cylindrotheca* | *fusiformis* |  | Diatom | ✓ (N) | - | 1. (Fischer *et al.*, 1999) |
| *Chaetoceros* | *Sp.* | CCK09 | Diatom | ✓ (N) | - | (Miyagawa-Yamaguchi *et al.*, 2011) |
| *Fistulifera* | *sp.* | JPCC DA0580-A | Diatom | ✓ (N) |  | (Muto *et al.*, 2013) |
| *Fragilariopsis* | *cylindrus* | CCMP 1102 | Diatom | - | ✓(pending, JGI) |  |
| *Navicula* | *saprophila* | NAVICl | Diatom | ✓ (N) | - | (Dunahay *et al.*, 1995) |
| *Phaeodactylum* | *tricornutum* | CCMP2561 (genome), and University of Texas Culture Collection, strain 646 | Diatom | 1. ✓ (N, C) | ✓ | 1. (Apt *et al.*, 1996; Zaslavskaia *et al.*, 2001; Bowler *et al.*, 2008; Xie *et al*., 2014) |
| *Pseudo-nitzia* | *multiseries* | CLN-47 | Diatom | - | ✓(pending, JGI) |  |
| Thalassiosira | oceanica | CCMP1005 | Diatom | - | ✓ | (Lommer *et al.*, 2012) |
| *Thalassiosira* | *pseudonana* | CCMP 1335 (genome) | Diatom | ✓ (N) | ✓ | (Poulsen *et al.*, 2006; Armbrust *et al.*, 2004) |
| *Thalassiosira* | *weissflogii* | - | Diatom | ✓ (N) | - | (Falciatore *et al.*, 1999) |
| *Aurantiochytrium* | *limacinum* | MH0186 | heterokont | ✓ (N) | ✓(pending, JGI) | (Sakaguchi *et al.*, 2012) |
| *Aureococcus* | *anaphagefferens* |  | heterokont | - | ✓ | (Gobler *et al.*, 2011) |
| *Nannochloropis* | *sp.* | W2J3B | heterokont | ✓ (N)* | - | (Killian *et al*., 2011) |
| *Nannochloropis* | *gaditana* | CCMP526 | heterokont | ✓ (N) | ✓ | (Radakovits *et al.*, 2012) |
| *Nannochloropsis* | *oceanica* | CCMP1779 | heterokont | ✓ (N) | ✓ | (Vieler *et al.*, 2012) |
| *Nannochloropsis* | *oculata* | (Droop) D. J. Hibberd | heterokont | ✓ (N) | - | (Chen *et al.*, 2008) |
| *Parietichytrium* | sp. | TA04Bb | heterokont | ✓ (N) | - | (Sakaguchi *et al.*, 2012) |
| *Schizochytrium* | sp. TIO1101 and sp. SEK 579 | - | heterokont | ✓ (N) | - | (Cheng *et al.*, 2012; Sakaguchi *et al.*, 2012) |
| *Thraustochytrium* | *aureum* | ATCC 34304 | heterokont | ✓ (N)* | - | (Sakaguchi *et al.*, 2012) |
| Amphidinium | sp., | - | Dinophyta | ✓ (N) | - | (te and Miller, 1998) |
| Symbiodinium | microadriaticum | c. XAMA (fla) Sc1 | Dinophyta | ✓ (N) | - | (te and Miller, 1998) |
| Symbiodinium | minutum | - | Dinophyta | ✓ (N) | ✓ | (Shoguchi *et al.*, 2013; Mungpakdee *et al.*, 2014) |
| *Ectacarpus* | *siliculosus* | (Dillwyn) Lyngbye | Phaeophyta | - | ✓ | (Cock *et al.*, 2010) |
| *Laminaria* | *japonica* | F003 and M007 | Phaeophyta | ✓ (N) | - | (Jiang *et al.*, 2003) |
| *Bigelowiella* | *natans* | - | Chlorarachniophyta | - | ✓ | (Curtis *et al.*, 2012) |
| *Lotharella* | *amoebiformis* | - | Chlorarachniophyta | ✓ (N) | - | (Hirakawa *et al.*, 2008) |
| Lotharella | oceanica | CCMP622 | Chlorarachniophyta | - | ✓ | (Tanifuji *et al.*, 2014) |
| *Euglena* | *gracilis* | - | Euglenozoa | ✓ (C) | - | (Doetsch *et al.*, 2001) |

**Table S1**. Overview of microalgae that are transformable and/or have a published genome (key: *homologous recombination reported).

***References***

**Abe, J., Hori, S., Tsuchikane, Y., Kitao, N., Kato, M. and Sekimoto, H.** (2011) Stable nuclear transformation of the *Closterium peracerosum-strigosum-littorale* complex. *Plant Cell Physiol.*, **52**, 1676–85.

**Anila, N., Chandrashekar, A., Ravishankar, G.A. and Sarada, R.** (2011) Establishment of *Agrobacterium tumefaciens* -mediated genetic transformation in *Dunaliella bardawil*. *Eur. J. Phycol.*, **46**, 36–44.

**Apt, K.E., Grossman, A.R. and Kroth-Pancic, P.G.** (1996) Stable nuclear transformation of the diatom *Phaeodactylum tricornutum*. *MGG Mol. Gen. Genet.*, **252**, 572–579.

**Armbrust, E.V., Berges, J.A., Bowler, C., et al.** (2004) The genome of the diatom *Thalassiosira pseudonana*: ecology, evolution, and metabolism. *Science*, **306**, 79–86.

**Barbier, G., Oesterhelt, C., Larson, M. D., Halgren, R. G., Wilkerson, C., Garavito, R. M., … Weber, A. P. M.** (2005). Comparative genomics of two closely related unicellular thermo-acidophilic red algae, *Galdieria sulphuraria* and *Cyanidioschyzon merolae*, reveals the molecular basis of the metabolic flexibility of *Galdieria sulphuraria* and significant differences in carbo. *Plant Physiology*, **137**, 460–74.

**Bhattacharya, D., Price, D.C., Chan, C.X., et al.** (2013) Genome of the red alga *Porphyridium purpureum*. *Nat. Commun.*, **4**, 1941.

**Blanc, G., Duncan, G., Agarkova, I., et al.** (2010) The *Chlorella variabilis* NC64A genome reveals adaptation to photosymbiosis, coevolution with viruses, and cryptic sex. *Plant Cell*, **22**, 2943–55.

**Boynton, J., Gillham, N., Harris, E., et al.** (1988) Chloroplast transformation in *Chlamydomonas* with high velocity microprojectiles. *Science.*, **240**, 1534–1538.

**Bowler, C., Allen, A.E., Badger, J.H., et al.** (2008) The *Phaeodactylum* genome reveals the evolutionary history of diatom genomes. *Nature*, **456**, 239–44.

**Cha, T.S., Yee, W. and Aziz, A.** (2012) Assessment of factors affecting Agrobacterium-mediated genetic transformation of the unicellular green alga, *Chlorella vulgaris*. *World J. Microbiol. Biotechnol.*, **28**, 1771–9.

**Chen, H.L., Li, S.S., Huang, R. and Tsai, H.-J.** (2008) Conditional production of a functional fish growth hormone in the transgenic line of *Nannochloropsis oculata* (eustigmatophyceae). *J. Phycol.*, **44**, 768–776.

**Chen, Y., Wang, Y., Sun, Y., Zhang, L. and Li, W.** (2001) Highly efficient expression of rabbit neutrophil peptide-1 gene in *Chlorella ellipsoidea* cells. *Curr. Genet.*, **39**, 365–70.

**Cheng, R., Ma, R., Li, K., Rong, H., Lin, X., Wang, Z., Yang, S. and Ma, Y.** (2012) *Agrobacterium tumefaciens* mediated transformation of marine microalgae *Schizochytrium*. *Microbiol. Res.*, **167**, 179–86..

**Chow, K.-C. and Tung, W.L.** (1999) Electrotransformation of *Chlorella vulgaris*. *Plant Cell Rep.*, **18**, 778–780.

**Cock, J.M., Sterck, L., Rouzé, P., et al.** (2010) The *Ectocarpus* genome and the independent evolution of multicellularity in brown algae. *Nature*, **465**, 617–21.

**Corellou, F., Schwartz, C., Motta, J.-P., Djouani-Tahri, E.B., Sanchez, F. and Bouget, F.-Y.** (2009) Clocks in the green lineage: comparative functional analysis of the circadian architecture of the picoeukaryote *Ostreococcus*. *Plant Cell*, **21**, 3436–49.

**Cui, Y., Jiang, P., Wang, J., Li, F., Chen, Y., Zheng, G. and Qin, S.** (2011) Genetic transformation of *Platymonas* (*Tetraselmis*) *subcordiformis* (Prasinophyceae, Chlorophyta) using particle bombardment and glass-bead agitation. *Chinese J. Oceanol. Limnol.*, **30**, 471–475.

**Cui, Y., Qin, S. and Jiang, P.** (2014) Chloroplast transformation of *Platymonas* (*Tetraselmis*) *subcordiformis* with the bar gene as selectable marker. A. Webber, ed. *PLoS One*, **9**, e98607.

**Curtis, B.A., Tanifuji, G., Burki, F., et al.** (2012) Algal genomes reveal evolutionary mosaicism and the fate of nucleomorphs. *Nature*, **492**, 59–65.

**Dawson, H.N., Burlingame, R. and Cannons, A.C.** (1997) Stable Transformation of *Chlorella* : Rescue of Nitrate Reductase-Deficient Mutants with the *Nitrate Reductase Gene*. *Curr. Microbiol.*, **35**, 356–362.

**Derelle, E., Ferraz, C., Rombauts, S., et al.** (2006) Genome analysis of the smallest free-living eukaryote *Ostreococcus tauri* unveils many unique features. *Proc. Natl. Acad. Sci. U. S. A.*, **103**, 11647–52.

**Doetsch, N.A., Favreau, M.R., Kuscuoglu, N., Thompson, M.D. and Hallick, R.B.** (2001) Chloroplast transformation in *Euglena gracilis*: splicing of a group III twintron transcribed from a transgenic psbK operon. *Curr. Genet.*, **39**, 49–60.

**Dunahay, T.G., Jarvis, E.E. and Roessler, P.G.** (1995) Genetic transformation of the diatoms *Cyclotella cryptica* and *Navicula saprophila1*. *J. Phycol.*, **31**, 1004–1012.

**El-Sheekh, M.M.** (1999) Stable Transformation of the Intact Cells of *Chlorella Kessleri* with High Velocity Microprojectiles. *Biol. Plant.*, **42**, 209–216.

**Falciatore, A., Casotti, R., Leblanc, C., Abrescia, C. and Bowler, C.** (1999) Transformation of Nonselectable Reporter Genes in Marine Diatoms. *Mar. Biotechnol. (NY).*, **1**, 239–251.

**Feng, S., Xue, L., Liu, H. and Lu, P.** (2009) Improvement of efficiency of genetic transformation for *Dunaliella salina* by glass beads method. *Mol. Biol. Rep.*, **36**, 1433–9.

**Fischer, H., Robl, I., Sumper, M. and Kroger, N.** (1999) Targeting and covalent modification of cell wall and membrane proteins heterologously expressed in the diatom *Cylindrotheca fusiformis* (Bacillariophyceae). *J. Phycol.*, **35**, 113–120.

**Gan, S.Y., Qin, S., Othman, R.Y., Yu, D. and Phang, S.M.** (2003) Transient expression of lacZ in particle bombarded *Gracilaria changii* (Gracilariales, Rhodophyta). *J. Appl. Phycol.*, **15**, 351–353.

**Gao, C., Wang, Y., Shen, Y., Yan, D., He, X., Dai, J. and Wu, Q.** (2014) Oil accumulation mechanisms of the oleaginous microalga *Chlorella protothecoides* revealed through its genome, transcriptomes, and proteomes. *BMC Genomics*, **15**, 582.

**Geng, D., Wang, Y. and Wang, P.** (2003) Stable expression of hepatitis B surface antigen gene in *Dunaliella salina* (Chlorophyta). , **15**, 451–456.

**Gobler, C.J., Berry, D.L., Dyhrman, S.T., et al.** (2011) Niche of harmful alga *Aureococcus anophagefferens* revealed through ecogenomics. *Proc. Natl. Acad. Sci. U. S. A.*, **108**, 4352–7.

**Guo, S.-L., Zhao, X.-Q., Tang, Y., Wan, C., Alam, M.A., Ho, S.-H., Bai, F.-W. and Chang, J.-S.** (2013) Establishment of an efficient genetic transformation system in Scenedesmus obliquus. *J. Biotechnol.*, **163**, 61–8

**Gutiérrez, C.L., Gimpel, J., Escobar, C., Marshall, S.H. and Henríquez, V.** (2012) Chloroplast genetic tool for the green microalgae *Haematococcus pluvialis* (Chlorophyceae, Volvocales). *J. Phycol.*, **48**, 976–983.

**Hallmann, A., Rappel, A. and Sumper, M.** (1997) Gene replacement by homologous recombination in the multicellular green alga *Volvox carteri*. *Proc. Natl. Acad. Sci.*, **94**, 7469–7474.

**Hirakawa, Y., Kofuji, R. and Ishida, K.** (2008) Transient transformation of a chlorarachniophyte alga, *Lotharella amoebiformis* (Chlorarachniophyceae), with *UID A* and *EGFP* reporter genes. *J. Phycol.*, **44**, 814–820.

**Hori, K., Maruyama, F., Fujisawa, T., et al.** (2014) Klebsormidium flaccidum genome reveals primary factors for plant terrestrial adaptation. *Nat. Commun.*, **5**, 3978.

**Hsieh, H.-J., Su, C.-H. and Chien, L.-J.** (2012) Accumulation of lipid production in *Chlorella minutissima* by triacylglycerol biosynthesis-related genes cloned from Saccharomyces cerevisiae and Yarrowia lipolytica. *J. Microbiol.*, **50**, 526–34.

**Huang, X., Weber, J.C., Hinson, T.K., Mathieson, A.C. and Minocha, S.C.** (1996) Transient Expression of the GUS Reporter Gene in the Protoplasts and Partially Digested Cells of *Ulva lactuca* L. (Chlorophyta). *Bot. Mar.*, **39**..

**Imamura, S., Hagiwara, D., Suzuki, F., Kurano, N. and Harayama, S.** (2012) Genetic transformation of *Pseudochoricystis ellipsoidea*, an aliphatic hydrocarbon-producing green alga. *J. Gen. Appl. Microbiol.*, **58**, 1–10.

**Jiang, P., Qin, S. and Tseng, C.K.** (2003) Expression of the lacZ reporter gene in sporophytes of the seaweed Laminaria japonica (Phaeophyceae) by gametophyte-targeted transformation. *Plant Cell Rep.*, **21**, 1211–6.

**Kathiresan, S., Chandrashekar, A., Ravishankar, G.A. and Sarada, R.** (2009) *Agrobacterium* -mediated transformation in the green alga *Haematococcus pluvialis* (chlorophyceae, volvocales). *J. Phycol.*, **45**, 642–649..

**Kilian, O., Benemann, C.S.E., Niyogi, K.K. and Vick, B.** (2011) High-efficiency homologous recombination in the oil-producing alga *Nannochloropsis* sp. *Proc. Natl. Acad. Sci. U. S. A.*, **108**, 21265–9

**Kindle, K.L.** (1989) Stable nuclear transformation of *Chlamydomonas* using the *Chlamydomonas* gene for nitrate reductase. *J. Cell Biol.*, **109**, 2589–2601.

**Kurtzmann and Cheney** (1991) Direct gene transfer and transient gene expression in a marine red alga using the biolistic method. *J. Phycol.*, **27**, 42.

**Lapidot, M., Raveh, D., Sivan, A., Arad, S.M. and Shapira, M.** (2002) Stable chloroplast transformation of the unicellular red alga *Porphyridium* species. *Plant Physiol.*, **129**, 7–12.

**Lerche, K. and Hallmann, A.** (2013) Stable nuclear transformation of *Eudorina elegans*. *BMC Biotechnol.*, **13**, 11..

**Lerche, K. and Hallmann, A.** (2009) Stable nuclear transformation of *Gonium pectorale*. *BMC Biotechnol.*, **9**, 64.

**Lerche, K. and Hallmann, A.** (2014) Stable nuclear transformation of *Pandorina morum*. *BMC Biotechnol.*, **14**, 65.

**Liu, H., Yu, W., Dai, J., Gong, Q., Yang, K. and Zhang, Y.** (2003) Increasing the transient expression of GUS gene in *Porphyra yezoensis* by 18S rDNA targeted homologous recombination. *J. Appl. Phycol.*, **15**, 371–377.

**Liu, J., Sun, Z., Gerken, H., Huang, J., Jiang, Y. and Chen, F.** (2014) Genetic engineering of the green alga *Chlorella zofingiensis*: a modified norflurazon-resistant phytoene desaturase gene as a dominant selectable marker. *Appl. Microbiol. Biotechnol.*, **98**, 5069–79..

**Liu, L., Wang, Y., Zhang, Y., Chen, X., Zhang, P. and Ma, S.** (2013) Development of a new method for genetic transformation of the green alga *Chlorella ellipsoidea*. *Mol. Biotechnol.*, **54**, 211–9..

**Lommer, M., Specht, M., Roy, A.-S., et al.** (2012) Genome and low-iron response of an oceanic diatom adapted to chronic iron limitation. *Genome Biol.*, **13**, R66.

**Lozano, J.-C., Schatt, P., Botebol, H., Vergé, V., Lesuisse, E., Blain, S., Carré, I.A. and Bouget, F.-Y.** (2014) Efficient gene targeting and removal of foreign DNA by homologous recombination in the picoeukaryote *Ostreococcus*. *Plant J.*, **78**, 1073–83.

**Maruyama, M., Hor�kov�, I., Honda, H., Xing, X., Shiragami, N. and Unno, H.** (1994) Introduction of foreign DNA into *Chlorella saccharophila* by electroporation. *Biotechnol. Tech.*, **8**, 821–826.

**Matsuzaki, M., Misumi, O., Shin-I, T., et al.** (2004) Genome sequence of the ultrasmall unicellular red alga *Cyanidioschyzon merolae* 10D. *Nature*, **428**, 653–7.

**Mei, K., Su-juan, W., Yao, L., Da-leng, S. and Cheng-kui, Z.** (1998) Transient expression of exogenous gus gene in *Porphyra yezoensis* (Rhodophyta). *Chinese J. Oceanol. Limnol.*, **16**, 56–61.

**Merchant, S.S., Prochnik, S.E., Vallon, O., et al.** (2007) The *Chlamydomonas* genome reveals the evolution of key animal and plant functions. *Science*, **318**, 245–50.

**Minoda, A., Sakagami, R., Yagisawa, F., Kuroiwa, T. and Tanaka, K.** (2004) Improvement of culture conditions and evidence for nuclear transformation by homologous recombination in a red alga, *Cyanidioschyzon merolae* 10D. *Plant Cell Physiol.*, **45**, 667–71.

**Miyagawa-Yamaguchi, A., Okami, T., Kira, N., Yamaguchi, H., Ohnishi, K. and Adachi, M.** (2011) Stable nuclear transformation of the diatom *Chaetoceros* sp. *Phycol. Res.*, **59**, 113–119.

**Moreau, H., Verhelst, B., Couloux, A., et al.** (2012) Gene functionalities and genome structure in *Bathycoccus prasinos* reflect cellular specializations at the base of the green lineage. *Genome Biol.*, **13**, R74.

**Mungpakdee, S., Shinzato, C., Takeuchi, T., et al.** (2014) Massive gene transfer and extensive RNA editing of a symbiotic dinoflagellate plastid genome. *Genome Biol. Evol.*, **6**, 1408–22.

**Muto, M., Fukuda, Y., Nemoto, M., Yoshino, T., Matsunaga, T. and Tanaka, T.** (2013) Establishment of a genetic transformation system for the marine pennate diatom *Fistulifera* sp. strain JPCC DA0580--a high triglyceride producer. *Mar. Biotechnol. (NY).*, **15**, 48–55.

**Nakamura, Y., Sasaki, N., Kobayashi, M., et al.** (2013) The first symbiont-free genome sequence of marine red alga, Susabi-nori (*Pyropia yezoensis*). J. Kroymann, ed. *PLoS One*, **8**, e57122.

**Niu, Y.F., Zhang, M.H., Xie, W.H., Li, J.N., Gao, Y.F., Yang, W.D., Liu, J.S. and Li, H.Y.** (2011) A new inducible expression system in a transformed green alga, *Chlorella vulgaris*. *Genet. Mol. Res.*, **10**, 3427–34..

**Ooijen, G. van, Knox, K., Kis, K., Bouget, F.-Y. and Millar, A.J.** (2012) Genomic transformation of the picoeukaryote *Ostreococcus tauri.* *J. Vis. Exp.*, e4074.

**Palenik, B., Grimwood, J., Aerts, A., et al.** (2007) The tiny eukaryote *Ostreococcus* provides genomic insights into the paradox of plankton speciation. *Proc. Natl. Acad. Sci. U. S. A.*, **104**, 7705–10.

**Pombert, J.-F., Blouin, N.A., Lane, C., Boucias, D. and Keeling, P.J.** (2014) A lack of parasitic reduction in the obligate parasitic green alga *Helicosporidium*. J. Heitman, ed. *PLoS Genet.*, **10**, e1004355.

**Poulsen, N., Chesley, P.M. and Kröger, N.** (2006) Molecular genetic manipulation of the diatom *Thalassiosira pseudonana* (Bacillariophyceae). *J. Phycol.*, **42**, 1059–1065.

**Price, D.C., Chan, C.X., Yoon, H.S., et al.** (2012) *Cyanophora paradoxa* genome elucidates origin of photosynthesis in algae and plants. *Science*, **335**, 843–7.

**Prochnik, S.E., Umen, J., Nedelcu, A.M., et al.** (2010) Genomic analysis of organismal complexity in the multicellular green alga *Volvox carteri*. *Science*, **329**, 223–6.

**Radakovits, R., Jinkerson, R.E., Fuerstenberg, S.I., Tae, H., Settlage, R.E., Boore, J.L. and Posewitz, M.C.** (2012) Draft genome sequence and genetic transformation of the oleaginous alga *Nannochloropis gaditana*. *Nat. Commun.*, **3**, 686.

**Rathod, J.P., Prakash, G., Pandit, R. and Lali, A.M.** (2013) *Agrobacterium*-mediated transformation of promising oil-bearing marine algae *Parachlorella kessleri*. *Photosynth. Res.*, **118**, 141–6.

**Read, B.A., Kegel, J., Klute, M.J., et al.** (2013) Pan genome of the phytoplankton *Emiliania* underpins its global distribution. *Nature*, **499**, 209–13..

**Saei, A.A., Ghanbari, P. and Barzegari, A.** (2012) *Haematococcus* as a promising cell factory to produce recombinant pharmaceutical proteins. *Mol. Biol. Rep.*, **39**, 9931–9..

**Sakaguchi, K., Matsuda, T., Kobayashi, T., et al.** (2012) Versatile transformation system that is applicable to both multiple transgene expression and gene targeting for Thraustochytrids. *Appl. Environ. Microbiol.*, **78**, 3193–202.

**Schiedlmeier, B., Schmitt, R., Muller, W., Kirk, M.M., Gruber, H., Mages, W. and Kirk, D.L.** (1994) Nuclear transformation of *Volvox carteri.* *Proc. Natl. Acad. Sci.*, **91**, 5080–5084.

**Shoguchi, E., Shinzato, C., Kawashima, T., et al.** (2013) Draft assembly of the *Symbiodinium minutum* nuclear genome reveals dinoflagellate gene structure. *Curr. Biol.*, **23**, 1399–408..

**Sørensen, I., Fei, Z., Andreas, A., Willats, W.G.T., Domozych, D.S. and Rose, J.K.C.** (2014) Stable transformation and reverse genetic analysis of *Penium margaritaceum*: a platform for studies of charophyte green algae, the immediate ancestors of land plants. *Plant J.*, **77**, 339–51.

**Sun, X.-M., Tang, Y.-P., Meng, X.-Z., Zhang, W.-W., Li, S., Deng, Z.-R., Xu, Z.-K. and Song, R.-T.** (2006) Sequencing and analysis of a genomic fragment provide an insight into the Dunaliella viridis genomic sequence. *Acta Biochim. Biophys. Sin. (Shanghai).*, **38**, 812–20.

**Tan, C., Qin, S. and Zhang, Q.** (2005) Establishment of a micro-particle bombardment transformation system for *Dunaliella saliva*. , **43**, 361–365.

**Tanifuji, G., Onodera, N.T., Brown, M.W., Curtis, B.A., Roger, A.J., Ka-Shu Wong, G., Melkonian, M. and Archibald, J.M.** (2014) Nucleomorph and plastid genome sequences of the chlorarachniophyte *Lotharella oceanica*: convergent reductive evolution and frequent recombination in nucleomorph-bearing algae. *BMC Genomics*, **15**, 374.

**te, M.R. and Miller, D.J.** (1998) Genetic transformation of dinoflagellates (*Amphidinium* and *Symbiodinium*): expression of GUS in microalgae using heterologous promoter constructs. *Plant J.*, **13**, 427–435.

**Teng, C., Qin, S., Liu, J., Yu, D., Liang, C. and Tseng, C.** (2002) Transient expression of *lacZ* in bombarded unicellular green alga *Haematococcus pluvialis*. *J. Appl. Phycol.*, **14**, 497–500.

**Vannerum, K., Abe, J., Sekimoto, H., Inzé, D. and Vyverman, W.** (2010) Intracellular localization of an endogenous cellulose synthase of *Micrasterias denticulata* (Desmidiales, Chlorophyta) by means of transient genetic transformation. *J. Phycol.*, **46**, 839–845.

**Vieler, A., Wu, G., Tsai, C.-H., et al.** (2012) Genome, functional gene annotation, and nuclear transformation of the heterokont oleaginous alga *Nannochloropsis oceanica* CCMP1779. D. Bhattacharya, ed. *PLoS Genet.*, **8**, e1003064.

**Wang, J., Jiang, P., Cui, Y., Deng, X., Li, F., Liu, J. and Qin, S.** (2010) Genetic transformation in *Kappaphycus alvarezii* using micro-particle bombardment: a potential strategy for germplasm improvement. *Aquac. Int.*, **18**, 1027–1034.

**Worden, A.Z., Lee, J.-H., Mock, T., et al.** (2009) Green evolution and dynamic adaptations revealed by genomes of the marine picoeukaryotes *Micromonas*. *Science*, **324**, 268–72.

**Xie, W.-H., Zhu, C.-C., Zhang, N.-S., Li, D.-W., Yang, W.-D., Liu, J.-S., Sathishkumar, R. and Li, H.-Y.** (2014) Construction of novel chloroplast expression vector and development of an efficient transformation system for the diatom *Phaeodactylum tricornutum*. *Mar. Biotechnol.,* **16**, 538–46.

**Zaslavskaia, L.A., Lippmeier, J.C., Kroth, P.G., Grossman, A.R. and Apt, K.E.** (2001) Transformation of the diatom *Phaeodactylum tricornutum* (Bacillariophyceae) with a variety of selectable marker and reporter genes. *J. Phycol.*, **36**, 379–386.

**Zorin, B., Grundman, O., Khozin-Goldberg, I., Leu, S., Shapira, M., Kaye, Y., Tourasse, N., Vallon, O. and Boussiba, S.** (2014) Development of a nuclear transformation system for Oleaginous Green Alga *Lobosphaera* (*Parietochloris*) *incisa* and genetic complementation of a mutant strain, deficient in arachidonic acid biosynthesis. M.-J. Virolle, ed. *PLoS One*, **9**, e105223.
