# Supplementary material for: Establishing Chlamydomonas reinhardtii as an industrial biotechnology host
Source: Plant J. 2015 Mar 8;82(3):532–46. doi: 10.1111/tpj.12781 (PMC4515103; doi:10.1111/tpj.12781)
Supplement: Supplementary file 3 [file tpj0082-0532-sd3.docx]

**Supporting Information Legends**

**Figure S1. Algal-biotechnological advancements from a phylogenetic perspective.** A schematic diagram of the eukaryotic tree of life (based on, (Dorrell and Smith, 2011)) displaying only super groups that contain photosynthetic lineages. The integrated heat map indicates the number of species in each phylogenetic grouping that i) are transformable (T), ii) have a published genome (G) (whereby superscript numbers indicate the actual number of species according to Table S1).

**Table S1**. Overview of microalgae that are transformable and/or have a published genome.
